# Parent–child interactive behavior in a German sample of parents with and without a mental illness: model replication and adaption of the Coding Interactive Behavior system

**DOI:** 10.3389/fpsyt.2024.1266383

**Published:** 2024-04-30

**Authors:** Julia Fahrer, Philipp Doebler, Klara Hagelweide, Pius Kern, Nora Nonnenmacher, Vanessa Seipp, Corinna Reck, Christina Schwenck, Sarah Weigelt, Anna-Lena Zietlow, Hanna Christiansen

**Affiliations:** ^1^ Clinical Child- and Adolescent Psychology, Department of Psychology, University of Marburg, Marburg, Germany; ^2^ Special Needs Educational & Clinical Child and Adolescent Psychology, Department of Psychology, Justus Liebig University Giessen, Gießen, Germany; ^3^ Department of Statistical Methods in the Social Sciences, Technical University Dortmund, Dortmund, Germany; ^4^ Department of Vision, Visual Impairments & Blindness, Faculty of Rehabilitation Science, Technical University Dortmund, Dortmund, Germany; ^5^ Center for Psychosocial Medicine, Heidelberg University Hospital, Institute of Medical Psychology, Heidelberg, Germany; ^6^ Department of Clinical Child and Adolescent Psychology, Faculty of Psychology, Ludwig-Maximilians-University München, Munich, Germany; ^7^ Clinical Child- and Adolescent Psychology, Department of Psychology, Technical University Dresden, Dresden, Germany

**Keywords:** parent-child interaction, Coding Interactive Behavior, CIB, parents with a mental illness, factorial structure, behavior observation, factor analysis, psychometric properties

## Abstract

Studies using observational measures often fail to meet statistical standards for both reliability and validity. The present study examined the psychometric properties of the Coding Interactive Behavior (CIB) System within a German sample of parent–child dyads. The sample consisted of 149 parents with and without a mental illness and their children [n experimental group (EG) = 75, n control group (CG) = 74] who participated in the larger Children of Mentally Ill Parents at Risk Evaluation (COMPARE) study. The age of the children ranged from 3 to 12 years (M = 7.99, SD = 2.5). Exploratory factor analysis supported a five-factor model of the CIB with items describing 1) parental sensitivity/reciprocity, 2) parental intrusiveness, 3) child withdrawal, 4) child involvement, and 5) parent limit setting/child compliance. Compared to international samples, the model was reduced by two independent dyadic factors. Testing for predictive validity identified seven items with predictive power to differentiate parental group membership. The CIB factors did not seem to be sufficiently sensitive to illustrate differences in interaction within a sample of parents with various mental illnesses. To apply the CIB to the described sample or similar ones in the future, additional measurement instruments may be necessary.

## 1 Introduction

Interactive behaviors are the central communication between parents and their children from birth onward (1). Visual contact, a friendly smile, or an affectionate touch from the parent usually builds a child’s first social experiences. The Coding Interactive Behavior (CIB) is a widely used tool for measuring parental, child, and dyadic interactive behavior (2). Especially in infancy and early childhood, children depend on their parents’ intuitive ability to recognize their behavioral signals and to respond to them promptly and appropriately (3–5). Therefore, the interactive behavior of parents with their children is of paramount importance for the development of the child (6). The content of the interaction must be adapted to the child’s cognitive, motor, and socioemotional developmental stages (1). Repeatedly, experienced parental behaviors are internalized by the child and contribute to the child’s perception of safety, empathy, emotion regulation, and moral development (1, 7). Parental interactive style within interactions is stable over time and should be foreseeable for the child (Feldman, 2010 (8)).

Parental sensitivity is a widely used construct to measure parental responsiveness to infant needs (9, 10) and is the core concept within the environment that promotes an infant’s attachment security (11). It consists of supportive parental behaviors such as parental receptivity, responsiveness, contingent reactions, and age-adapted stimulation, involvement, and communication to children’s signals and appears to be a core parental behavioral factor for both infant and child development (8, 12).

As children become more active in their interactions with their parents, dyadic reciprocity becomes an important behavioral expression alongside parental sensitivity. Dyadic reciprocity describes an interactive coordination of gaze, affect, vocalization, and touch of both partners within the interaction. The mutual regulation of affect in reciprocal interaction is necessary for the development of a child’s regulatory skills (13). In later childhood, the content of the dyadic interaction shifts from play to verbal dialogue.

Parental behavior is influenced by the respective culture of the interactive partners and their temperament and may be affected by possible parental illness, especially mental disorders (1). Sensitive maternal behavior toward offspring, whether infants, children, or adolescents, predicts socioemotional development, adaption (14, 15), and cognitive development (14, 16). A high amount of maternal warmth, low levels of punishing disciplinary actions, appropriate limit setting, and dyadic reciprocity within the interaction are associated with improved child self-regulation (17, 18) and resilience (13).

Given the central role of parent–child interactions in child development, it is clear that contextual factors such as cultural influences and parental mental health play an important role in shaping these interactions. Recognizing this, our study seeks to account for these influences by examining the psychometric properties of the CIB tool, thereby enhancing our understanding of parent–child interactions across contexts and ensuring the robustness of our research findings.

## 2 Background

### 2.1 Parent–child interaction in the context of parental mental illness

Parental interactive behavior can be impaired when a parent has a mental illness and may appear different from that of parents without a mental illness. The model of the transgenerational transmission of mental disorders (TTMDs) identifies parental hostility, rejection, low involvement, abuse, neglect, and lack of sensitivity as well as reduced child responsiveness and imitation as potential mechanisms of disorder transmission under the broad category of parent–child interaction (19, 20). A meta-analysis by Rodrigues et al. (21) points out that low parental sensitivity and child behavior problems are mutually reinforcing, with a stronger effect in older children. The parental ability to recognize the child’s behavioral signals and to respond promptly and adequately can be impaired by experiences of violence, abuse (22, 23), war (24), and different parental mental illnesses (25–29).

For instance, mothers with depression show more disengagement, are less responsive to their offspring’s distress and social signals, talk less with their children, tend to avoid eye contact, and are more easily irritated (30, 31). They also provide less affectionate touch, need more time to react to shifts in the child’s behavior, have difficulty in determining an appropriate level of stimulation for the child, and prevail less reciprocity in the interaction (30, 31). For parents with an anxiety disorder, findings appear to be more heterogeneous regarding low parental sensitivity due to methodological issues in primary studies (e.g., different anxiety disorders, no clinical interviews, and different paradigms) (29, 32, 33). Parents with anxiety disorders show increased controlling behavior and intrusiveness, and interactions appear to be driven by the parent’s agenda and plans rather than the child’s. In addition, it appears that parents with anxiety disorders have difficulty estimating the adequate intensity of the interaction, tend to overstimulate their children, and thus disregard the child’s signals (1, 30). Mothers with schizophrenia show less sensitivity, increased intrusive behaviors, more self-centeredness, and withdrawal behavior, and their behavior appears to be even more impaired than that of mothers with bipolar disorder or depression (34). Mothers with post-traumatic stress disorder (PTSD) show less supportive behavior and sensitivity and have difficulty recognizing their child’s affective state and behavior. They respond inadequately, and, as a result, their children show more withdrawing behavior (24, 35, 36).

The stated findings offer a brief overview of the many existing studies on parental, especially maternal, interactive behavior, predominantly using the Coding Interactive Behavior when a parent has a mental illness. While most studies focus on maternal mental illness in early childhood, little is known about its effect across the age span of middle childhood (37). A recent meta-analysis of parent–child interactions (38) reported that the mean age of the children was 44 months, and 94% of the primary studies assessed maternal interactive behavior. Cross-lagged analyses show that sensitivity plays an important role in middle childhood and adolescence as well (12). Both mothers and fathers are primary caregivers within a family, but parent–child interactions have been studied primarily in mother–child dyads (39). Moreover, most studies examining the impact of parental mental illness look at one specific disorder rather than various mental illnesses. This raises the question of whether a specific parental mental illness exhibits a unique behavioral profile or whether parents with a mental illness present a similar clinical picture across diagnostic categories and in contrast to parents without a mental illness. Parental interactive behavior appears to be of great importance for child development and is influenced by parental mental illness. The composition of constructs describing interactive behavior is also influenced by culture.

### 2.2 Parent–child interaction and cultural sensitivity

Every culture has its own set of shared values, norms, beliefs, and behaviors that are considered normative in one culture or society, but not necessarily in another. Cultural beliefs and specific behaviors appear to be stable over time and are communicated to new members of a culture. This accounts for parenting behavior as well, as it maintains cultural conceptions (40). Parental behavior has a direct impact on infant behavior through repeated exchanges and interpersonal relatedness of experiences (30). During the postpartum period, parental behavior appears to be more similar across societies but becomes more diverse and culturally sensitive as children grow up (1). Cultural norms and attitudes shape a parent’s behavior to be consistent with overarching cultural goals and values (41).

For instance, European and American mothers use more suggestions than commands to structure their child’s behavior, in contrast to Puerto Rican mothers who use more immediate cues such as commands, physical manipulation, positioning, and restrictions to attract and draw their child’s attention and guide their play (42). German parents are often characterized by a more distal parental style (43). Verbal communication and focused attention are more prevalent than physical contact. German parents encourage their child’s curiosity and creativity to promote autonomy, independence, and cognitive competencies (44) and perceive their child as an equal interactive partner (45). Furthermore, physical manipulation of children is an indicator of relationship disturbances in Western societies when observed in mother–child interactions with children older than 1 year. However, it can also be observed in father–child interactions and is considered normal because it is categorized as “rough-and-tumble” play that promotes the father–child bond (1, 46, 47). In summary, there are cultural and gender differences in the occurrence of parental behaviors in interactions. In addition, there appears to be a need for an objectifiable tool to classify behavior as well as a valid, comprehensive measurement model.

### 2.3 The Coding Interactive Behavior system

The CIB is an observable, macro-analytic measure of parent–child interactive behavior. The CIB is a global coding scheme for “free-play” interactions in dyads that takes into account the behavior of both interacting partners. The CIB uses developmental goal-adapted paradigms, such as free play for infants and preschoolers or a joint discussion of a pre-defined topic (e.g., planning a fun day together) at school-aged children, to assess human behavior. Depending on the age of the child, it comprises up to 42 items (**Appendix A**), of which 21 address the parent’s behavior, 16 assess the child’s behavior, five are dyadic codes, and two are additional overall codes (44 in total). Behaviors are rated on 9-point Likert-type scales, where 5 is a strong expression of behavior and 1 is a weak expression (2). The CIB codes are comprised of eight factors, sometimes called composites: *parental sensitivity*, *intrusiveness*, and *limit setting*; *child engagement*–*involvement*, *withdrawal*, and *compliance*; *dyadic reciprocity*; and *negative states*. Further, the CIB contains items that are central to a construct and other items that are part of a construct in some cultures but not in others or at certain stages of development. Parental acknowledgment is an important component of the parental sensitivity construct across ages and cultures, whereas parental affectionate touch is an essential component of the sensitivity construct in some cultures (1, 2). The interrater reliability of the CIB appears to be substantial (48), and the internal consistency of the different composites ranges between adequate and good (α = 0.72–0.95) in different samples (1, 2, 6, 48).

The CIB is a widely used tool, including samples of premature infants and their mothers (49), clinically referred infants (50), parents with a mental illness (51), and biological and socioemotional risk factors (6, 52). The CIB has been used in Western societies, such as Israel (53), Germany (23, 29), France (54), and Denmark (55), and in non-Western societies, including Palestinian families in Ramallah and the West Bank (41); the CIB appears to be sensitive toward cultural variation (53).

The psychometric properties are reported in several studies stating measures of reliability (6, 48, 56), but information on factorial validity is often lacking (57). In line with Steenhoff et al. (55), we were not able to identify any validation of the CIB within a sample of fathers, with the exception of the study conducted by the respective authors (55, 57). The postulated factor structure has only been validated and verified in a French sample of newborns and their mothers (54) and for the three parental factors within a well-resourced Danish sample of 5-year-olds and their parents without mental illness (55). The Danish study was able to identify the three parental factors for mothers and five paternal factors based on a reduced set of items. To our knowledge, there has been no independent validation and verification of either the measurement model or the factorial structure of the CIB in addition to the two studies mentioned. As the paper by Viaux-Savelon et al. (54) focused on newborns and the paper by Steenhoff et al. (55) on 5-year-olds, a validation of the factorial structure within a sample of German mothers and fathers with and without mental disorders and their children across a broader age range is lacking. Moreover, the former paper (55) only reported Cronbach’s alpha, missing further measures to assert the factorial structure.

### 2.4 Aims

In order to analyze group differences between parents with and without a mental illness, it is important to assess whether the theoretically formulated constructs can be identified within the present sample. Further, it is important to assess how the constructs are composed depending on the specific culture and the age range of the children. Finally, comparing the interactive behavior of parents with different mental illnesses to healthy controls is an important step in understanding the impact of mental illness on parenting behavior and child outcomes and in developing interventions to support families affected by mental illness.

This study is the first to examine the psychometric properties of the CIB within a sample of German parents with different mental disorders as well as healthy control parents and their children aged 3 to 12 years. Specifically, the aims were to investigate a) the item properties and b) the construct validity, with an emphasis on exploring the dimensional structure of the postulated composites. In this regard, exploratory factor analysis was conducted to investigate whether the latent factor structures of parents replicated the postulated CIB parenting constructs. Further, we aimed to investigate c) the reliability and d) the interrater reliability of the instrument. The present study aims to provide the psychometric properties to enable further substantive analysis.

## 3 Method

The Children of Mentally Ill Parents at Risk Evaluation (COMPARE)-family study is a prospective multicenter, confirmatory, randomized controlled phase III trial with two parallel arms (58), funded by the German Federal Ministry of Education and Research (BMBF), providing cognitive behavioral therapy for parents with a mental illness. For more information, see Stracke et al. (58), Christiansen et al. (19), and Zietlow et al. (59). The present COMPARE-interaction study within the COMPARE-family project is a subproject of the larger COMPARE-interaction (59) and COMPARE-family projects (19).

### 3.1 Participants

The participants in the interaction study consisted of a subsample of parent–child dyads recruited for the larger COMPARE-family study (19, 58) and a control group (CG) without mental illness. Families met the inclusion criteria for the interaction study if a) parent and child agreed to participate in a videotaped, semi-structured play paradigm; b) children were between the ages of 3 and 12; c) families had sufficient knowledge of the German language; and d) parents were seeking treatment and met diagnostic criteria for a mental disorder according to DSM-5 and children did not have a mental disorder requiring urgent treatment to meet the inclusion criteria for the experimental group (EG). If the families who participated in the COMPARE-family study did not provide their consent to be videotaped, they were not able to participate in the interaction study. To meet the inclusion criteria for the CG, both parent and child were required not to present with any mental illness, and parents were required to report that they had never been treated for or diagnosed with a mental illness. Participants were assessed between 2018 and 2021 at Philipps University Marburg, Justus Liebig University Giessen, and Technical University Dortmund.

Data were collected on 75 children and 60 parents in the EG and 74 children and 59 parents in the CG. An overview of the sociodemographic characteristics is provided in **Table 1**. In the EG, 46 parents participated with one child, 13 with two children, and one parent with three children. In the CG, 48 parents participated with one child, seven parents with two children, and four parents with three children.

**Table 1:** Demographics of EG and CG.

	EG (n dyads = 75)	CG (n dyads = 74)	Mann–Whitney U-test
Child characteristics			
Sex: female	39 (52%)	31 (41.89%)	
Sex: male	36 (48%)	32 (43.23%)	
Mean age in years (SD)	7.48 (2.39)	8.5 (2.60)	W = 1,800.500, p = 0.02
Missing background information		11 (14.88%)	
Index patient’s characteristics			
Sex: female	50 (81.97%)	51 (86.44%)	
Mean age in years (SD)	38.75 (5.77)	41.83 (5.48)	W = 977, p = 0.003
SES (SD)	4.76 (.89)	6.41 (.63)	W = 104.50, p < 0.001
Cultural resources	4.91	5.68	W = 629.5, p = 0.001
Migration background	13 (21.31%)	4 (6.78%)	

SD, standard deviation; SES, socioeconomic status; EG, experimental group; CG, control group.

The Shapiro–Wilk test was carried out to control for the distribution of demographic variables (60), and the Mann–Whitney test was used to control for group comparability regarding background characteristics and symptom severity (61). Furthermore, the socioeconomic status (SES) of children of parents with a mental illness was lower than that of controls (W = 104.50, p < 0.001). However, when looking at representative data of children and adolescents in Germany, the SES of both groups can be classified as low (62). To assess the SES of both groups, occupational status and net household income were converted into numbers between 1 and 7 according to the scales used in the second wave of the KiGGS study (62), and the mean of both values was calculated. Families with a migrant background were underrepresented in the study in both the EG and the CG (63). Children in the EG showed higher scores on the internalizing subscale of the Child Behavior Checklist (CBCL), and parents in the EG showed higher Brief Symptom Inventory–Global Severity Index (BSI–GSI) scores (see **Table 2** for an overview of parent and child group differences). Participation in the study was voluntary for both groups, with families in the CG receiving a financial incentive. Families in the EG received gold standard CBT treatment according to the COMPARE-family protocol (58). Additional information on parental diagnosis is provided in the **Appendix**.

**Table 2:** Independent samples t-test of CBCL and BSI.

	Test	Statistic	df	p	Location parameter	SE difference
CBCL Total Problem Score	Student	2.791	145	0.003	6.095	2.184
CBCL Externalizing Behavior	Student	2.695	145	0.004	2.731	1.013
CBCL Internalizing Behavior	Student	4.868	145	<0.001	4.200	0.863
BSI GSI	Student	9.926	147	<0.001	36.529	3.680

For all tests, the alternative hypothesis specifies that group EG is greater than group CG. For Student’s t-test, location parameter is given by mean difference. For the Mann–Whitney test, location parameter is given by the Hodges–Lehmann estimate.

CBCL, Child Behavior Checklist; BSI, Brief Symptom Inventory; GSI, Global Severity Index; EG, experimental group; CG, control group.

### 3.2 Measure

In the EG, parental diagnosis as well as potential child disorders were assessed using the Diagnostic Interview of Mental Disorders for parents and children (DIPS and Kinder-DIPS) (62, 64) and the Structured Interview for Preschool Ages (SIVA) (65) for children under the age of 6. In both the EG and the CG, parental psychopathology was assessed using the BSI (66), and child psychopathology was assessed using the German version of the CBCL-parent version (67). Due to the comparability of the groups, only BSI and CBCL scores are reported in the present article.

#### 3.2.1 Child Behavior Checklist

The CBCL/6-18 (parent report) (68) was used to describe internalizing and externalizing behaviors in children age 6 years and older. The CBCL/1^1/2^-5 (parent report) (69) was used to characterize internalizing and externalizing behaviors in children under 6 years of age. The standardized behavior scales quantify children’s and adolescents’ emotional and behavioral difficulties over the past 6 months. The CBCL/6-18 comprises 118 items, and the CBCL/1^1/2^-5 comprises 99 items, each rated on a 3-point Likert-type scale ranging from 0 (*never or not true*) to 2 (*often or very true*). Items referring to rule-breaking and aggressive behavior were aggregated into an externalizing subscale. Items describing anxious/depressed, withdrawn/depressed, and somatic components were aggregated into an internalizing subscale. The CBCL has shown high test–retest reliability, criterion, and construct validity (70, 71). In the present study, the internal consistency of the total scale was α = 0.75.

#### 3.2.2 The Brief Symptom Inventory

The BSI (66) is a self-report questionnaire for adolescents and adults that assesses the subjective impairment concerning somatic and psychological symptoms (66). It comprises 53 items, rated on 5-point Likert-type scales ranging from 0 (*not at all*) to 4 (*very much*). The items refer to nine primary symptom scales and three global indices that depict the global burden, such as the GSI. The internal consistency of the total scales for our sample was α = 0.91.

#### 3.2.3 The Coding Interactive Behavior

The CIB is a global coding scheme for the analysis of behavior observations of dyads (2). The CIB enables the rating of specific behaviors and affective states of each interaction partner within a dyad, as well as an overall dyadic impression (2). The CIB appears to be sensitive to cultural variation (53), parental mental illness (51), biological and socioemotional risk factors (6, 52), and the effects of interventions (48, 72). The CIB is used from the newborn stage to adolescence with adapted coding manuals for the different age groups, providing manuals for newborns (2–36 months), preschoolers (3–6 years), school-aged children (6–12 years), and adolescents (2). The CIB is a 44-item global coding scheme that provides eight theoretically derived composites (48, 52, 53, 73–76), of which three composite scores with 22 items depict parental behaviors and affective states: *Parent Sensitivity*, *Parent Intrusiveness*, and *Parent Limit Setting*. Another three composites of 16 items are child-related: *Child Social Involvement*, *Withdrawal*, and *Compliance to Parent*. Two additional composites of five items refer to the dyadic behaviors and states: *Dyadic Reciprocity* and *Dyadic Negative States* (1, 2). Feldman (1) reported a sufficient model fit for the composites *Parent Sensitivity*, *Parent Intrusiveness*, *Child Social Involvement*, and *Child Negative Affect* [χ^2 =^ 56.12, p = 0.18, goodness-of-fit index (GFI) = 0.94, adjusted goodness-of-fit index (AGFI) = 0.93, normed fit index (NFI) = 0.92, root mean square error of approximation (RMSEA) = 0.03].

### 3.3 Procedure

This study was approved by the Ethical Committees of Philipps University Marburg, Justus Liebig University Giessen, and Technical University Dortmund, Germany. The recruitment for the EG consisted of electronic and paper flyers as well as posters that were distributed in clinics and private practices, mental health hospitals, schools, bus commercials, topic-related readings, and Facebook. Furthermore, the research team contacted almost every local youth care institution that supports families and children in order to raise awareness of the project and to request that information about the project be forwarded to suitable families. The families in the CG were recruited as a convenience sample with electronic and paper flyers as well as posters that were distributed at schools, flea markets, and private practices and on Facebook.

All participating parents and children of school age gave their written informed consent prior to or on the date of the assessment. In the case of shared child custody, both parents provided written informed consent. Parents completed the CBCL and BSI questionnaires online. Parent–child interaction observations were carried out and videotaped by graduate students and undergraduate assistants at the respective universities in laboratory settings. The semi-structured play paradigm invited the parents to spend time together with their children as they usually do. Therefore, a set of several toys for free play situations was provided. Videos were pseudonymized afterward and exchanged between reliable raters to ensure the blindness of the raters toward group allocation and parental diagnosis. A subset of 21 videos was blindly coded by all reliable raters enrolled in a Ph.D. or postdoctoral program.

### 3.4 Data analysis

#### 3.4.1 Item properties

Data from 149 dyads were used for the analysis of the item properties. The means and standard deviation of every item of the CIB were assessed. **Table 3** shows all items as they are traditionally assigned to the original factors according to Feldman’s model (training provided by Ruth Feldman in 2020). For the items child *Fatigue* and *Parent Depressed Mood*, this was performed in their original form as well as after reversing them as suggested in the training provided by the research group of Feldman. In order to investigate how well the items differentiate between individuals in our sample, their discriminatory power was analyzed.

**Table 3:** Original theoretical model.

Parent composites:	
Parental sensitivity (9 items)	Acknowledging (core item), Elaborating, Parent Gaze, Positive Affect, Vocal Appropriateness, Appropriate Range of Affect, Resourcefulness, Praising, Affectionate Touch, Parent Supportive Presence
Parental intrusiveness (6 items)	Overriding (core item), Forcing, Parent Negative Affect/Anger, Hostility, Parent Anxiety, Criticizing
Parent limit setting (3 items)	Consistency of Style, On-Task Persistence, Appropriate Structure/Limit Setting
Child composites	
Child involvement (9 items)	Child Initiation (core item), Child Gaze, Child Positive Affect, Child Affection to Parent, Alert, Fatigue (Revised), Child Vocalization, Competent Use of the Environment, Creative Symbolic Play
Child withdrawal (4 items)	Negative Emotionality, Withdrawal, Emotion Lability, Child Avoidance of Parent
Child compliance (3 items)	Child Compliance to Parent, Child Reliance on Parent for Help, Child On-Task Persistence
Dyadic composites	
Dyadic reciprocity (3 items)	Reciprocity (core item), Adaptation-Regulation, and Fluency
Dyadic negative states (2 items)	Constriction and Tension

Core items are expected to be included in composites across contexts (CIB Training provided by R. Feldman, 2020).

The item discrimination and reliability were reported based on our newly specified model. The item child *Fatigue* was inverted as suggested by the research group of Ruth Feldman within the training they provided in 2020. Regarding the item properties, an item difficulty of P_i_ of between 5 and 95 appears to be achievable if one aims to depict the whole spectrum of characteristic features and even wants to differentiate between persons with extreme expressions of characteristic values. Furthermore, discriminatory power >0.4 to 0.7 was considered good (77).

#### 3.4.2 Construct validity

In the current study, we conducted an exploratory factor analysis (EFA) with the whole sample consisting of both the CG and EG.

##### 3.4.2.1 Model adaption

We performed an EFA (78) using the R software package EFAtools [version 0.3.1 (79)]. The minimum and maximum number of factors to be extracted must be specified. We based this decision on a scree test (80) and a parallel analysis (81). In line with Preacher and MacCallum (82), we considered factor loadings smaller than 0.30 to be too small to be relevant and dropped them from further analysis. Therefore values .3 and above are displayed in bold letters. We assumed that the identified factors would correlate, as composites have been found to correlate in previous research (8). We decided to start with an EFA, as this is the first study examining the dimensional structure of the CIB within a sample of parents with various mental illnesses as well as including the item *Parent Depressed Mood*, and we aimed to explore the data without making any prior assumptions. We performed the EFA with the total sample of 149 dyads using oblimin, promax, and bifactor rotations and a minimal residual resolution (minres) using principal axis factoring (PAF) to calculate the model fit.

An EFA can provide evidence of whether current CIB practice mostly based on samples of dyads with infants from diverse cultural backgrounds is justified in a sample of preschool and school-age German-speaking children–parent dyads. Further, it can especially help to approach the question of dimensionality. By using several rotations, we can sketch avenues for further work on an appropriate measurement model, which would need to be based on a larger sample. For applications with relatively pure scores for subscales, we employed promax, oblimin, and bifactor (83).

#### 3.4.3 Predictive validity

Since the CIB items have repeatedly been demonstrated to be indicative of group differences, especially in the context of mental health, item scores and EFA factor scores were validated based on the CG and the EG. Two different methods were employed: (1) three multivariate analyses of variance (MANOVAs), each with the factor group (CG *vs.* EG) based on the factor scores of oblimin, promax, and oblique bifactor. The scores were deemed predictive if the MANOVAs were significant, indicating some latent mean group differences. For an appropriate test procedure, Friedrich and Pauly’s (84) MANOVA for heteroscedastic data was used. (2) A logistic regression with a (relaxed) least absolute shrinkage and selection operator (LASSO) was used to predict group membership. All factor scores and all item scores were used. Tuning of the LASSO was by 10-fold cross-validation. Based on a recent critical review of labeling conventions of area under the curve (AUC) values by de Hond et al. (85), the AUC was interpreted.

#### 3.4.4 Reliability

Cronbach’s alpha was used to calculate the internal consistency of the different composites presented in the factor analysis. A minimum value of 0.80 was considered good. Nevertheless, the number of items included was taken into account when interpreting the alpha coefficient (86).

#### 3.4.5 Interrater reliability

Reliable raters were trained to code with a substantial agreement of at least 80%. In order to achieve this, training was first provided by the research group of Ruth Feldman, and in a next step within the group of raters. A subset of 27 videos out of the 149 parent–child interactions was chosen for reliability training, with the aim of covering all age groups present in the study, as the initial training predominantly covered children of newborn and infant age. Videos were individually coded by each rater, and discrepancies in the ratings were solved through discussion to achieve a deep, shared understanding of the postulated items within the research group.

To calculate interrater agreement, a subset of 20% of the videos was randomly chosen and coded by four raters at the different study centers. The intraclass correlation coefficient (ICC) was calculated using a one-way random-effects model for multiple raters, and the average of k ratings was selected for absolute agreement (87) using the R psych software package [version 2.1.3 (88)]. Absolute agreement for the items *Compliance to Parent*, *Reliance on Parent for Help*, *Child Fatigue Recoded*, *Alert*, *Parent Anxiety*, and *Parent Appropriate Range of Affect* was moderate (0.50–0.75). The ICC for all remaining 38 items was good (0.75–0.90) to excellent (>0.90).

## 4 Results

### 4.1 Item properties

First, the analysis of the item properties was performed based on the model suggested by Feldman and her research group. However, the EFA of the CIB scales revealed a different five-factor structure. Therefore, the analyses were re-run using this revised model. The report of the results regarding item properties was restricted to these second analyses.

#### 4.1.1 Parental scales

The item difficulty of all parental items ranged from P_i_ = 20.13 (*Forcing*) to P_i_ = 92.62 (*Consistency of Style* and *Parental Depression Recoded*). Only the *Parent Anxiety item* achieved an item difficulty greater than P_i_ = 95 and was therefore excluded from further analysis.

The means of the items of the postulated *Sensitivity* scale ranged from *M* = 1.18 (*SD* = 0.5; *Affectionate Touch*) to *M* = 4.37 (*SD* = 0.68; *Parent Gaze*), with a possible maximum of 5. The discriminatory powers ranged from *r_it_*_(_ *_i_*_)_ = −0.02 (*Affectionate Touch*) to *r_it_*_(_ *_i_*_)_ = 0.68 (*Elaborating* and *Acknowledging*). The discriminatory power of all items was considered good (>0.40), except for the items *Praising* and *Affectionate Touch*, which were considered poor (<0.20).

The means of the items on the postulated *Intrusiveness* scale ranged from *M* = 1.01 (*SD* = 0.06; *Forcing*) to *M* = 1.63 (*SD* = 0.83; *Overriding*), with a possible minimum of 1 and maximum of 5.0. The discriminatory powers ranged from *r_it_*_(_ *_i_*_)_ = 0.0 (*Forcing*) to *r_it_*_(_ *_i_*_)_ = 0.98 (*Overriding*). The items *Parent Negative Affect* and *Hostility* showed weak discriminatory power (<0.40), and the items *Forcing* and *Parent Anxiety* even displayed poor discriminatory power close to 0. The remaining items of the *Intrusiveness Scale* showed good discriminatory power.

The means of the items on the postulated *Limit Setting* scale ranged from *M* = 4.61 (*SD* = 0.7; *Appropriate Structure*) to *M* = 4.63 (*SD* = 0.61; *Consistency of Style*), with a possible maximum of 5.0. The discriminatory powers ranged from *r_it_*_(_ *_i_*_)_ = 0.34 (*Parent On-Task Persistence*) to *r_it_*_(_ *_i_*_)_ = 0.64 (*Parent Appropriate Structure*_)_. The discriminatory power was weak (<0.40) for one item (*Parent On-Task Persistence*) and good for the remaining items.

#### 4.1.2 Child scales

The item difficulty of all child items ranged from P_i_ = 22.55 (*Child negative affect* and *Withdrawal*) to P_i_ = 94.77 (*Child Gaze*). Only the item *Fatigue Recoded* achieved an item difficulty above P_i_ = 95 (p = 98.46) and was therefore excluded from further analysis.

Means of the items of the postulated *Child Involvement* Scale ranged from *M* = 2.16 (*SD* = 1.38; *Creative–Symbolic Play*) to *M* = 4.28 (*SD* = 0.68; *Competent Use of the Environment*), excluding *Fatigue* M = 1.08 (SD = 0.3) and *Fatigue Recoded* M = 4.92 (*SD* = 0.3). The discriminatory powers ranged from *r_it_*_(_ *_i_*_)_ = 0.47 (*Child Gaze*) to *r_it_*_(_ *_i_*_)_ = 0.73 (*Child Positive Affect*). The discriminatory power was good, except for the item *Fatigue Recoded*, which showed weak discriminative power *r_it_*_(_ *_i_*_)_ = 0.16.

The means of the items of the postulated *Withdrawal* Scale ranged from *M* = 1.09 (*SD* = 0.29; *Avoidance of Parent*) to *M* = 1.14 (*SD* = 0.34; *Withdrawal*). The item difficulty and discriminatory powers ranged from P_i_ = 1.08, *r_it_*_(_ *_i_*_)_ = 0.07 (*Avoidance of Parent*) to P_i_ = 1.14, *r_it_*_(_ *_i_*_)_ = 0.27 (*Withdrawal*). All items on the scale showed weak discrimination.

Means of the items of the postulated *Compliance to Parent* scale ranged from *M* = 1.69 (*SD* = 0.82; *Reliance on Parent for Help*) to *M* = 4.72 (*SD* = 0.48; *On-Task Persistence*). The discriminatory powers ranged from weak *r_it_*_(_ *_i_*_)_ = 0.01 (*Reliance on Parent for Help*) to good *r_it_*_(_ *_i_*_)_ = 0.63 (*Compliance to Parent*).

#### 4.1.3 Dyadic scales

Item difficulty of all dyadic items was within the spectrum of 5 ≤ P_i_ ≤ 20, e.g., 80 ≤ 95.

Means of the items of the postulated *Dyadic Reciprocity* scale ranged from *M* = 3.84 (*SD* = 0.86; *Dyadic Reciprocity*) to *M* = 3.94 (*SD* = 0.74; *Adaptation-Regulation*). The discriminatory powers ranged from *r_it_*_(_ *_i_*_)_ = 0.65 (*Adaptation-Regulation*) to *r_it_*_(_ *_i_*_)_ = 0.79 (*Fluency*).

Means of the items of the postulated *Dyadic Negative States* scale ranged from *M* = 1.19 (*SD* = 0.43; *Tension*) to *M* = 1.61 (*SD* = 0.75; *Constriction*). The discriminatory powers ranged from *r_it_*_(_ *_i_*_)_ = −0.43 (*Tension*) to *r_it_*_(_ *_i_*_)_ = −0.71 (*Constriction*).

### 4.2 Construct validity

#### 4.2.1 Preliminary analysis

The data for the assumption of univariate and multivariate normality were tested with respect to skewness and kurtosis. For skewness and kurtosis, the items *Parent Depressed Mood* and *Child Fatigue* were imputed in their original form, as recoded items would only result in reversed skewness values. Overall, 26 out of 44 items showed values of skewness greater than I1I, and 23 items showed kurtosis greater than I1I. For all 26 items, skewness was significant, and 23 items showed significant kurtosis.

#### 4.2.2 EFA

Bartlett’s test of sphericity, which tests the significance of the item correlations within a correlation matrix, resulted in *χ* ^2^ (721) = 4,119.98, p < 0.00, indicating that factor analysis appears appropriate (89). The Kaiser–Meyer–Olkin (KMO) test revealed an overall KMO = 0.87 and was therefore considered meritorious, indicating that the strength of the relationship between the items was high (90). The KMO for the items *Praising*, *Affectionate Touch*, *On-Task Persistence*, *Criticizing*, child *Persistence*, and *Creative Play* were below 0.5. Therefore, they were excluded from further analysis. The empirical Kaiser–Guttman criterion (KGC) suggested the extraction of five factors. The scree plot revealed five eigenvalues before the substantial drop. In the parallel analysis, there were six eigenvalues before the point of intersection of the present data with the data line simulated from random data. However, the sixth eigenvalue was very close to the randomly generated plot. The Velicer MAP test (91, 92) achieved a minimum of 0.02 with six factors, and the empirical Bayesian information criterion (BIC) achieved a minimum of −2,330.4 with five factors when using promax, oblimin, or bifactor rotation. Based on these results, the *goodness-of-fit indices* of a one-factor baseline model and a five-factor model were compared with the reduced item pool as proposed in the KMO analysis (**Table 4**) (93, 94).

**Table 4:** Model fit exploratory factor analysis (minres PAF estimator).

Model	*χ* ^2^	df	*χ* ^2^/df	RMSEA [90% CI]	TLI	BIC
1 Factor	4,119.99***	741	5.56	0.12 [0.11; 0.13]	0.47	−1,885.86
5 Factors	1,045.38	556	1.88	0.08 [0.07; 0.08]	0.80	−1,736.82

χ^2^, Satorra–Bentler scaled chi-square; CFI, comparative fit index; RMSEA, root mean square error of approximation; CI, confidence interval; TLI, Tucker–Lewis index; BIC, Bayesian information criterion; minres, minimal residual resolution; PAF, principal axis factoring.

***p < 0.001.

A model fit is considered to be good if *χ* ^2^/*df* ≤ 2 or as acceptable if *χ* ^2^/*df* ≤ 3 (95). The inspection of the fit indices of the different EFA models indicated that the reduced five-factor solution when removing the items *Parent Anxiety*, *Praising*, *Affectionate Touch*, *child Fatigue Recoded*, and *Reliance on Parent for Help* represented the data the best and resulted in a good model fit of *χ* ^2^/*df* = 1.88, Tucker–Lewis index (TLI) = 0.80 appeared mediocre (TLI ≥ 0.8), and RMSEA = 0.08 appeared to be acceptable (RMSEA ≤ 0.08). It was decided to continue with the reduced five-factor model. **Table 5** shows the factor loadings of the generated model using promax rotation. Please compare the **Appendix** for the EFA using oblimin and bifactor rotations.

**Table 5:** Factor loadings on the 5-factor EFA model (promax) (n = 149).

	Factor 1 “Parent Sensitivity/Reciprocity”	Factor 2 “Parent Intrusiveness”	Factor 3 “Child Involvement”	Factor 4 “Parent Limit Setting/Child Persistence”	Factor 5 “Child Withdrawal”
PposAffect	0.993	**0.043**	**−0.081**	**−0.170**	**−0.097**
Penthusiasm	0.966	**0.127**	**−0.027**	**−0.144**	**−0.098**
Pdeprrec	0.903	**0.207**	−0.408	**0.029**	**−0.241**
PAppRangeAff	0.876	**−0.060**	**−0.005**	**−0.212**	**0.166**
PRessourcefulness	0.744	**−0.044**	**−0.041**	**0.152**	**0.210**
DyFluency	0.726	**0.015**	**0.133**	**0.110**	**−0.008**
PVocApp	0.658	**−0.141**	**−0.077**	**0.147**	**−0.003**
DyConstriction	−0.589	**0.107**	**−0.125**	**−0.012**	**0.207**
PSuppPres	0.573	**−0.279**	**0.281**	**−0.071**	**0.034**
DyReciprocity	0.551	**−0.174**	**0.221**	**0.111**	**−0.023**
Pgaze	0.533	**0.023**	**−0.035**	0.386	**0.173**
PAcknowledge	0.519	−0.400	**0.125**	**−0.072**	**0.183**
PElaborate	0.511	**−0.226**	**0.127**	**−0.020**	**0.122**
DyTension	**−0.103**	0.863	**0.097**	**0.166**	**0.213**
PCriticizing	**−0.070**	0.728	**0.261**	**0.080**	**0.066**
PHost	**−0.082**	0.692	**0.216**	**0.116**	**0.268**
Pled	**0.138**	0.621	**−0.178**	**−0.198**	**−0.139**
POverriding	**0.065**	0.537	−0.327	**−0.138**	**−0.074**
ChLed	**−0.001**	−0.512	0.346	**0.170**	**0.182**
DyAdaptation	0.394	−0.413	**0.207**	**−0.066**	**0.084**
ChInitiation	**−0.149**	**0.120**	0.741	**0.258**	**−0.185**
ChVocalization	**0.077**	**0.060**	0.627	**−0.103**	**−0.139**
ChPosAff	0.426	**0.159**	0.619	**0.003**	**−0.277**
ChWithdrawal	**0.114**	**−0.115**	−0.595	−0.327	**0.080**
ChAltert	**0.264**	**0.209**	0.540	**−0.054**	**0.035**
ChCreatPlay	**−0.128**	**−0.101**	0.520	**−0.240**	**0.014**
ChAfftoPar	**0.113**	**0.084**	0.364	**−0.110**	−0.332
ChCompUse	**0.144**	**−0.153**	**0.242**	**0.063**	**0.052**
ChPers	**−0.142**	**0.195**	**−0.057**	0.806	**−0.021**
POnTaskPers	**−0.133**	**−0.120**	**−0.107**	0.648	**−0.061**
ChGaze	**0.188**	**0.182**	**0.057**	0.634	**0.091**
PAppStrukt	**0.026**	**−0.212**	**−0.107**	0.523	**−0.171**
ChCompliance	**0.120**	**−0.115**	**−0.056**	0.445	**−0.230**
PConsistency	**0.243**	**−0.153**	**−0.023**	**0.250**	**0.032**
PForcing	**−0.006**	**−0.085**	**0.002**	**−0.032**	0.637
ChNegAff	**0.000**	0.434	**−0.018**	**−0.027**	0.588
PNegAff	**−0.041**	0.526	**0.222**	**0.042**	0.582
ChLabile	**0.127**	**0.059**	**−0.148**	**−0.149**	0.539
ChAvoidance	**0.048**	**0.017**	**−0.201**	**0.010**	0.494

Applied rotation method is promax.

EFA, exploratory factor analysis.

In contrast to Feldman’s postulated eight-factor model, our EFA revealed a five-factor model. We identified factors related to traditional factors such as Parental Sensitivity/Reciprocity, Parental Intrusiveness, Child Involvement, Parent Limit Setting/Child Compliance, and Child Withdrawal, as shown in **Table 5**. However, the EFA model did not identify separate factors associated with the individual for parental, child, and dyadic behaviors. Within the EFA using bifactor rotation, we were able to identify a main factor. This was related to parental sensitivity, dyadic reciprocity, and child involvement. The other four factors were Parent Sensitivity, Parent Intrusiveness, Child Withdrawal, and Parent Limit Setting/Child Compliance (see **Appendix**).

In this study, we conducted EFA using promax, oblimin, and bifactor rotations to explore the underlying structure of the observed variables. Factors related to Parental Sensitivity/Reciprocity, Parental Intrusiveness, Child Involvement, Parent Limit Setting/Child Compliance, and Child Withdrawal were identified in both analyses (see **Table 6**). The **Appendix** presents the factor loadings, the proportion of variance explained, and factor correlations obtained from the oblimin rotation.

**Table 6:** Factors identified using different rotations for EFA.

	Promax	Oblimin
Factor 1	Parent Sensitivity/Reciprocity	Parent Sensitivity/Reciprocity
Factor 2	Parent Intrusiveness	Parent Intrusiveness/Child Withdrawal
Factor 3	Child Involvement	New positive dyadic factor
Factor 4	Parent Limit Setting/Child Compliance	Child Involvement
Factor 5	Child Withdrawal	Parent Limit Setting/Child Compliance

All analyses using oblimin rotation are displayed in the **Appendix**.

EFA, exploratory factor analysis.

The results of the rotated factor solution using promax rotation provide important insights into the underlying structure of the variables under consideration (**Table 7**). The proportion of variance explained by each factor provides valuable information about the relative importance of these factors in explaining the variability in the observed data.

**Table 7:** Eigenvalues, variance explained, and factor correlations for rotated factor solution using promax rotation.

Property	Factor_1	Factor_2	Factor_3	Factor_4	Factor_5
SS loadings	7.974	4.416	3.534	2.730	2.491
Proportion Var	0.204	0.113	0.091	0.070	0.064
Cumulative Var	0.204	0.318	0.408	0.478	0.542
Proportion explained	0.377	0.209	0.167	0.129	0.118
Cumulative proportion	0.377	0.586	0.753	0.882	1.000
Factor_1	1.000	−0.590	0.468	0.433	−0.227
Factor_2	−0.590	1.000	−0.390	−0.507	0.110
Factor_3	0.468	−0.390	1.000	0.417	0.049
Factor_4	0.433	−0.507	0.417	1.000	−0.007
Factor_5	−0.227	0.110	0.049	−0.007	1.000

Factor 1, Parent Sensitivity/Reciprocity; Factor 2, Parent Intrusiveness; Factor 3, Child Involvement; Factor 4, Parent Limit Setting; Child Persistence; Factor 5 = Child Withdrawal.

Factor 1, identified as Parent Sensitivity/Reciprocity, emerged as the most influential factor, explaining 37.7% of the variance in the data, followed by Factor 2, representing Parent Intrusiveness, which explained 20.9% of the variance. Factor 3, which captured Child Involvement, accounted for 16.7% of the variance, while Factors 4 and 5, which represented Parent Limit Setting/Child Persistence and Child Withdrawal, explained 12.9% and 11.8% of the variance, respectively. The cumulative proportion of variance explained by all five factors was 100%, indicating that together they comprehensively accounted for the variability in the observed variables. In addition, the correlations between the factors provide insight into the interrelationships among the underlying constructs. Factors 1 and 3 showed a moderate positive correlation (0.468), suggesting a relationship between Parent Sensitivity/Reciprocity and child involvement. Conversely, Factors 1 and 2 showed a negative correlation (−0.590), suggesting a possible trade-off between Parent Sensitivity/Reciprocity and Parent Intrusiveness.

#### 4.2.3 Predictive validity

None of the three MANOVAs were significant. The logistic regression reached a maximal AUC during cross-validation of approximately 0.67, with seven items—Parent Positive Affect, Parent Depression Recoded, Parent Appropriate Range of Affect, Parent On-Task Persistence, Child Compliance, Child Affection to Parent, and Dyadic Constriction—influencing the prediction. There was, hence, weak evidence of the predictive usefulness of some of the CIB items, but none of the factor scores contributed to the prediction. An AUC value of 0.67 indicated that the model was moderately effective at distinguishing between the two classes, resp. groups.

#### 4.2.4 Reliability

Reliability analysis was based on the original model formulated by Feldman (1998) (2) (Model A) and the newly specified Model B, which lacked the independent dyadic factors Reciprocity and Tension. We computed Cronbach’s α coefficients based on our model as a measure of internal consistency. The results are displayed in **Table 8**. All α coefficients were slightly better in Model B, except for the α coefficients for the factor *Involvement*. For *Child Withdrawal*, internal consistency was questionable with very low values of Cronbach’s α (see **Table 8** for details). The identified α coefficients are in contrast to the α coefficients identified by Keren and Feldman (96) in young children, which ranged from 0.85 to 0.95 and were based on the traditional measure model.

**Table 8:** Cronbach’s α for the traditional Model A formulated by R. Feldman and the newly specified Model B retrieved from the EFA.

Cronbach’s α	Model A	Model B
Factors		
Sensitivity/(Reciprocity)	0.92	0.92
Intrusiveness	0.63	0.83
Limit Setting/(Compliance)	0.58	0.76
Involvement	0.75	0.65
Withdrawal	0.56	0.58
Compliance	0.24	
Dyadic Reciprocity	0.87	
Dyadic negative State	0.70	

Cronbach’s α for shared factors sensitivity/reciprocity and Limit Setting/Compliance retrieved from Model B are in contrast to those of the traditional model. The α values are based on the EFA using promax rotation.

EFA, exploratory factor analysis.

## 5 Discussion

The present study evaluated the factor structure of the CIB in a sample of German parents with and without a mental illness. The study adds to the previous research by providing a comprehensive analysis of the psychometric properties of the CIB. The authors first considered the item properties, followed by the construct validity, with a focus on examining the dimensional structure, the measure invariance, and the reliability of the instrument. The respective objectives are shown in italics and bold in the following section.

The analysis of the *item properties* resulted in a 32-item CIB version and suggested the elimination of 10 items within the present sample. The application of the KMO revealed that seven items—*Parent Forcing*, *Praising*, *Affectionate Touch*, *On-Task Persistence*, *Criticizing*, child *Persistence*, and *Creative Play*—should be removed in order to best represent the data, as they were not fit for factorial analysis (90). These findings are similar to those of Steenhoff et al. (55), who performed the EFA for mothers and fathers separately. The KMO in their study suggested the elimination of the parental items *Forcing*, *Depressed Mood*, *Praising*, *Affectionate Touch*, and *Object Oriented* for mothers. For fathers, the KMO indicated the removal of *Overriding*, *Anxiety*, *Criticizing*, and *Affectionate Touch*.

In our study and based on our sample, the removed items show a few weaknesses: *Reliance on Parent for Help*, *Praising*, and *Affectionate Touch* showed weak discriminatory power in the present sample. Starting with *Affectionate Touch*, the item showed a low mean, indicating that this behavior was rarely observed. This is not surprising, as *Affectionate Touch* has been described as a culturally sensitive item (1), and in parenting in Northern and Middle European countries, such as Germany or Denmark, less physical closeness/touching and more distal behaviors are observed (43), compared to countries in Southern Europe or Israel (97, 98), where the instrument was originally developed. Apart from that, parental Affectionate Touch tends to decrease as children grow older (99). According to Keller et al. (44), German parents place great value on a child’s autonomy and cognitive competence, and children are perceived as equal interactive partners from an early age (45). This might also provide an explanation for the weak discrimination of the item *Reliance on Parent for Help*.

Both the Danish mothers without any mental illness and the parents in our mixed German sample showed little praise for their children’s actions. However, Viaux-Savelon et al. (55) were able to identify maternal praise in their sample of mothers with and without a mental illness and their children up to 2 months of age. It can therefore be concluded that maternal or parental praising can be observed in Central European cultures but seems to be sensitive toward the children’s age, emerging in dyads with very young children.

Additionally, *Parent Anxiety* and *Child Fatigue Recoded* achieved weak discrimination and a high item difficulty, suggesting that they were too difficult. The poor discriminatory power and high item difficulty of *Parent Anxiety* are surprising, as one might expect to observe various expressions of anxious behaviors in the present sample. This is in contrast to the Danish study, which was able to keep the item in the model for mothers but not for fathers (55), and the French study for clinically and non-clinically referred mothers (54). One might assume a gender difference when aiming to investigate parental anxious behavior, which should be further investigated. Another possible explanation may be that one might expect multidimensionality, which calls for factorial analysis when items present with poor discriminatory power (77). Unlike the study by Steenhoff et al. (55), the items *Depressed Mood*, *Overriding*, and *Critique* were suitable to enter factorial analysis in our sample. This may be caused by the sample composition of parents with and without a mental illness.

The analysis of *construct validity* resulted in a five-factor model with the following factors: Parental Sensitivity/Reciprocity, Parental Intrusiveness, Child Involvement, Parental Limit Setting/Child Compliance, and Child Withdrawal. The analyses indicated that parent limit setting merges with the factor of child compliance and parental sensitivity merges with dyadic reciprocity. This is not surprising, as strong associations between the two factors have been described in the previous research. According to the paper by Stuart et al. (100), we were also unable to identify isolated dyadic factors. For example, sensitivity and reciprocity were loaded on a common factor. When utilizing the bifactor model, we identified a general dyadic factor that represents positive aspects of parent–child interactions and is associated with sensitivity.

The bifactor model provides a clearer understanding of the underlying structure of the CIB data by decomposing variance into general and specific factors. One potential future direction is to differentiate between dyads using the dyadic main factor from the bifactor model. However, further research is necessary to investigate the differences and possible advantages of a general factor model and the traditional eight-factor model.

Additionally, this raises the question of whether parental sensitive behavior within an interaction can be assessed independently of dyadic behavior and thus also of the child’s behavior. This statistical inseparability at the factor level raises exciting questions for future research projects, and possible practical implications should be investigated in more detail.

Previous studies identified intrusive behavior to be more prevalent within the interactions of parents with anxiety disorders (1, 30) and schizophrenia (34). We were able to identify a strong factor depicting intrusiveness with a good internal consistency (α = 0.80). As disregarding a child’s signals and needs, overriding parental behavior, has been described to be more prevalent in parents with a mental illness (1), an imputation of not only the factor *Intrusiveness* but also the item parental *Overriding* might be of interest in future analyses. *Overriding* is the main aspect of the factor *Intrusiveness*, and the single item can be used as an index of intrusive parenting behavior (101). Another possibility to capture negative aspects of interactions in families of parents with a mental illness may be to measure the absence of sensitivity rather than the presence of intrusiveness. The results of various studies of parental depression, anxiety disorders (30), and PTSD (24, 35, 36) emphasized low levels of sensitivity. As evidence of factorial validity is only reported in very few studies on behavior observation instruments (57), the assessment of the dimensionality of the theoretically driven composites using factor analysis is a special highlight of the present article.

The consistency of the five factors across both rotation methods enhances the robustness and generalizability of our findings, providing confidence in the stability of the identified factor structure. The use of both rotation methods provides a comprehensive examination of the data and allows for a robust interpretation of the factor structure. This approach enhances the validity and reliability of the findings and facilitates a deeper understanding of the relationships between the variables under study.

As observational studies often fail to meet statistical standards for both reliability and validity (102), a major advantage of the present study is to first examine the validity of the instrument used before considering other observation-specific components (e.g., reliability, task, and setting) (57, 103). The Child Withdrawal factor showed poor internal consistency. Unlike the study by Steenhoff et al. (2019) (55), which reported moderate internal consistency reliability for Parent *Limit Setting*, we were able to obtain alpha values above acceptable. Low alpha values can be caused by a small number of items, poor inter-relatedness, or heterogeneous constructs (104). Apart from the Danish study by Steenhoff et al. (55), which only assessed the parental constructs, this is the first independent study to present the internal consistency of the theoretically derived factors of the CIB within a sample of children aged 3 to 12 years, indicating that the factor *Child Withdrawal* should be used and interpreted with caution when studying the respective sample.

We depicted a strong interdependence between parental sensitivity and child involvement in the factor correlations. These factors have been associated with protective behavioral factors that promote child wellbeing (17, 18) and resilience (13) in previous studies.

The statistical analyses provide insights into the predictive validity of the CIB items in parent–child interactions. Although the three MANOVA tests were not significant, the logistic regression model showed a moderate AUC. This indicates a moderate level of effectiveness in distinguishing between the two groups or classes under consideration. Notably, seven specific items emerged as influential predictors in the logistic regression model: Parent Positive Affect, Parent Depression (recoded), Parent Appropriate Range of Affect, Parent On-Task Persistence, Child Compliance, Child Affection to Parent, and Dyadic Constriction. Against the background of various parental mental illnesses, the parental items all seem to address parental affect and appear to be able to distinguish well between the group affiliations within the present sample. This finding is of particular importance, as the present sample is the first with the various parental mental illnesses. The items Child Compliance and Child Affection to Parent are likely important aspects of parent–child interactions during middle childhood that can differentiate between the two groups. These items are developmentally significant and can provide valuable insight into the parent–child relationship. At the same time, it should be noted that children’s behavior is not solely determined by their own actions but also influenced by the behavior of their parents. Therefore, it can be assumed that parental and child effects within the interaction mutually influence each other. However, it must be said at this point that these findings cannot be generalized to other populations.

Although these findings suggest weak evidence of the predictive utility of some CIB items, it is important to note that none of the factor scores derived from exploratory factor analysis made a significant contribution to the prediction. Therefore, while the logistic regression model demonstrates moderate discriminative ability, the lack of significant contribution from factor scores implies a nuanced relationship between CIB items and predictive outcomes. These findings emphasize the intricate nature of interactions between parents and children and emphasize the necessity for additional research to clarify the specific mechanisms that underlie predictive validity in this area. The CIB factors did not seem to be sufficiently sensitive to illustrate differences in interaction within a sample of parents with various mental illnesses. Given that the CIB has historically been utilized for particular disorders, this discovery appears to be novel. To apply the CIB to the described sample or similar ones in the future, additional measurement instruments may be necessary.

To substantiate the robustness of our findings and further establish the reliability of the instrument, future research efforts should prioritize the expansion of the participant pool to capture a larger sample. Furthermore, the inclusion of additional psychometric quality criteria such as test–retest stability and the assessment of convergent and divergent validity will contribute to a more comprehensive validation framework for the instrument and thus improve its reliability and applicability in different contexts.

## 6 Limitations

The present study was mainly limited by the small sample size. According to Kline (105), the sample should comprise at least five participants per model parameter for factor analyses, which was not met by the present data. Observational studies require significant resources for data acquisition, compared to questionnaire studies. In the context of observational studies, our sample size is quite considerable. The present analysis therefore provides a first attempt to investigate the factorial validity of the CIB given the limitations of such observational tools. Additionally, the sample was not representative of the German population, such that, for example, few fathers participated in the present study. Similar challenges regarding the sample size, parental gender, and representativity have been reported by Steenhoff et al. (55), and future studies should aim for larger sample sizes, emphasizing the recruitment of fathers to enable group comparisons across parental gender. Within the CIB, two different coding manuals are used for children under and over the age of 6. Although this approach takes developmental differences into account, it introduces a potential confounding factor that can significantly affect the validity of the study. The presence of different manuals raises concerns about the generalizability and comparability of results across different age groups, which could affect the robustness of the validation study. Future research efforts in this area should explore methods that ensure more homogeneous age groups to improve the internal validity and reliability of the observed parent–child interactions.

The confirmatory factor analysis (CFA) and subsequent assessments of measure invariance were compromised by the challenging model fits ranging from poor to satisfactory. However, achieving a good model fit in a large model based on small samples has been described as challenging because common fit indices are sensitive to sample sizes (106). Most studies using behavior observations are limited by small sample sizes and often do not report model fit indices. This association should be assessed more precisely in further studies.

We included multiple children within one family in our study, resulting in a nested data structure, but did not perform multilevel analysis. This is a serious limitation of the present study. Ignoring this nested structure can lead to underestimation of the variability within the higher-level units and overestimation of the variability within the lower-level units. Further, we did not analyze associations between the specific parental diagnoses and interactive behavior, as this is beyond the scope of the present article. These various statistical possibilities should be exhausted in future analyses.

## 7 Conclusion

In conclusion, this study systematically evaluated the factor structure and psychometric properties of the CIB in a sample of German parents, both with and without a mental illness. The comprehensive analysis encompassed item properties, construct validity, and model fit in the context of confirmatory and exploratory factor analyses. The findings revealed the need for refinement in the CIB instrument, with the elimination of specific items that demonstrated weak discriminatory power or were culturally sensitive, reflecting nuances in parenting practices across different regions. Moreover, the identification of a statistical inseparability at the factor level raised intriguing questions for future research and emphasized the necessity of exploring these nuances for an improved understanding of parent–child interactions. While the study offered valuable insights into the factor structure and validity of the CIB, it also acknowledged limitations, including a relatively small sample size and challenges in achieving optimal model fits, which are common in observational studies.

In conclusion, this research contributes to the ongoing refinement of the CIB instrument and underscores the importance of cultural context, parental mental illness, and developmental considerations in understanding parent–child interactions.

## Data availability statement

The raw data supporting the conclusions of this article will be made available by the authors, without undue reservation.

## Ethics statement

The studies involving humans were approved by Ethics committee Philipps-University Marburg. The studies were conducted in accordance with the local legislation and institutional requirements. Written informed consent for participation in this study was provided by the participants’ legal guardians/next of kin.

## Author contributions

JF: Conceptualization, Data curation, Formal analysis, Investigation, Writing – original draft. VS: Writing – review & editing, Investigation. KH: Writing – review & editing, Investigation. NN: Writing – review & editing, Conceptualization. CR: Writing – review & editing, Conceptualization. CS: Conceptualization, Writing – review & editing, Funding acquisition, Methodology. SW: Writing – review & editing, Funding acquisition. A-LZ: Writing – review & editing, Conceptualization. HC: Writing – review & editing, Conceptualization, Funding acquisition, Methodology, Supervision. PD: Writing – review & editing, Formal analysis. PK: Writing – review & editing.
