# Supplementary material for: Parent–child interactive behavior in a German sample of parents with and without a mental illness: model replication and adaption of the Coding Interactive Behavior system
Source: Front Psychiatry. 2024 Apr 30;15:1266383. doi: 10.3389/fpsyt.2024.1266383 (PMC11091726; doi:10.3389/fpsyt.2024.1266383)
Supplement: Supplementary file 1 [file DataSheet_1.docx]

| Factor analysis results (oblimin) | | | | | |
| --- | --- | --- | --- | --- | --- |
|  | **Factor 1**  Parent Sensitivity/ Reciprocity | **Factor 2**  Parent Intrusiveness/ Child Withdrawal | **Factor 3** | **Factor 4**  Child Involvement | **Factor 5**  Parent limit setting/ Child Compliance |
| **PPosAffect** | 0.889 | *-0.108* | *-0.075* | *0.028* | *-0.083* |
| **Penthusiasm** | 0.861 | *-0.060* | *-0.118* | *0.080* | *-0.072* |
| **PAppRangeAff** | 0.799 | *0.089* | *0.163* | *0.005* | *-0.114* |
| **Pdeprrec** | 0.781 | *-0.201* | -0.364 | *-0.203* | *0.078* |
| **PRessourcefulness** | 0.691 | *0.131* | *0.194* | *-0.020* | *0.213* |
| **DyFluency** | 0.673 | *-0.007* | *0.078* | *0.189* | *0.156* |
| **PVocApp** | 0.616 | *-0.105* | *0.129* | *-0.007* | *0.208* |
| **PSuppPres** | 0.560 | *-0.082* | *0.337* | *0.263* | *0.009* |
| **DyConstriction** | -0.550 | *0.236* | *-0.035* | *-0.209* | *-0.064* |
| **DyReciprocity** | 0.535 | *-0.092* | *0.227* | *0.244* | *0.163* |
| **PAcknowledge** | 0.516 | *-0.030* | *0.457* | *0.077* | *0.026* |
| **Pgaze** | 0.503 | *0.136* | *0.139* | *-0.001* | *0.403* |
| **PElaborate** | 0.495 | *-0.001* | *0.301* | *0.107* | *0.051* |
| **PNegAff** | *-0.060* | 0.797 | *0.017* | *0.067* | *-0.018* |
| **ChNegAff** | *-0.031* | 0.723 | *0.005* | *-0.152* | *-0.063* |
| **DyTension** | *-0.155* | 0.617 | -0.469 | *0.080* | *0.044* |
| **PHost** | *-0.116* | 0.600 | *-0.279* | *0.157* | *0.019* |
| **PForcing** | *0.012* | 0.520 | *0.418* | *-0.177* | *-0.004* |
| **ChLabile** | *0.106* | 0.479 | *0.205* | *-0.271* | *-0.118* |
| **PCriticizing** | *-0.110* | 0.446 | -0.408 | *0.251* | *-0.025* |
| **ChAvoidance** | *0.041* | 0.412 | *0.206* | -0.302 | *0.028* |
| **ChLed** | *0.074* | *-0.039* | 0.605 | *0.239* | *0.216* |
| **Pled** | *0.050* | *0.148* | -0.602 | *-0.088* | *-0.247* |
| **POverriding** | *-0.014* | *0.146* | -0.550 | *-0.242* | *-0.182* |
| **DyAdaptation** | *0.407* | *-0.108* | 0.431 | *0.164* | *0.018* |
| **ChCompUse** | *0.162* | *0.001* | *0.233* | *0.206* | *0.084* |
| **ChInitiation** | *-0.100* | *0.002* | *0.062* | 0.712 | *0.191* |
| **ChPosAff** | *0.402* | *-0.095* | *-0.051* | 0.660 | *-0.003* |
| **ChVocalization** | *0.091* | *-0.007* | *0.072* | 0.587 | *-0.114* |
| **ChWithdrawal** | *0.070* | *-0.070* | *-0.088* | -0.564 | *-0.264* |
| **ChAltert** | *0.249* | *0.199* | *0.048* | 0.492 | *-0.065* |
| **ChCreatPlay** | *-0.091* | *0.045* | *0.216* | 0.416 | *-0.232* |
| **ChAfftoPar** | *0.103* | *-0.202* | *-0.138* | 0.413 | *-0.119* |
| **ChPers** | *-0.114* | *0.063* | *-0.098* | *0.017* | 0.712 |
| **POnTaskPers** | *-0.088* | *-0.129* | *0.079* | *-0.045* | 0.605 |
| **ChGaze** | *0.188* | *0.162* | *0.012* | *0.101* | 0.581 |
| **PAppStrukt** | *0.058* | *-0.274* | *0.080* | *-0.017* | 0.510 |
| **ChCompliance** | *0.133* | *-0.274* | *-0.011* | *0.050* | 0.431 |
| **PConsistency** | *0.246* | *-0.059* | *0.162* | *0.003* | *0.271* |

| Eigenvalues, Variance Explained, and Factor Correlations for Rotated Factor Solution using oblimin rotation | | | | | |  |
| --- | --- | --- | --- | --- | --- | --- |
| **Property** | **Factor 1**  Parent Sensitivity/ Reciprocity | **Factor 2**  Parent Intrusiveness/ Child Withdrawal | **Factor 3** | **Factor 4**  Child Involvement | **Factor 5**  Parent limit setting/ Child Compliance | |
| SS loadings | 7.498 | 3.693 | 3.552 | 3.510 | 2.891 | |
| Proportion Var | 0.192 | 0.095 | 0.091 | 0.090 | 0.074 | |
| Cumulative Var | 0.192 | 0.287 | 0.378 | 0.468 | 0.542 | |
| Proportion Explained | 0.355 | 0.175 | 0.168 | 0.166 | 0.137 | |
| Cumulative Proportion | 0.355 | 0.529 | 0.697 | 0.863 | 1.000 | |
| Factor_1 | 1.000 | -0.387 | 0.314 | 0.315 | 0.233 | |
| Factor_2 | -0.387 | 1.000 | -0.131 | -0.095 | -0.147 | |
| Factor_3 | 0.314 | -0.131 | 1.000 | 0.226 | 0.280 | |
| Factor_4 | 0.315 | -0.095 | 0.226 | 1.000 | 0.273 | |
| Factor_5 | 0.233 | -0.147 | 0.280 | 0.273 | 1.000 | |

**Factor analysis results (bifactor)**

F1 F2 F3 F4 F5

PForcing -.111 -.108 .021  **.603** -.020

POverridin **-.690** .250 .168 -.203 -.072

PAcknowled  **.751** .165 -.158  **.307** -.076

PElaborate  **.656** .198 -.053 .219 -.037

Pgaze  **.581** .274 .063 .237  **.303**

PPosAffect  **.639**  **.624** -.002 -.013 -.140

Pdeprrec  **.333**  **.706** -.055 -.195 .059

PNegAff **-.385** -.073  **.495**  **.443** .006

PHost **-.433** -.023  **.562** .119 .061

PVocApp  **.679**  **.365** -.091 .100 .117

PAppRangeA  **.595**  **.481** -.007 .240 -.178

PConsisten  **.443** .099 -.076 .103 .199

PRessource  **.653**  **.392** .013 .288 .117

POnTaskPer  **.303** -.095 -.088 -.004  **.529**

PAppStrukt  **.457** .002 -.162 -.080  **.428**

PCriticizi **-.389** .009  **.579** -.073 .024

Penthusias  **.610**  **.607** .075 -.029 -.127

PSuppPres  **.859** .195 -.028 .161 -.099

ChGaze  **.415** .078 .200 .116  **.490**

ChPosAff  **.784** .136  **.359** -.205 -.089

ChNegAff **-.513** .005  **.327**  **.451** -.018

ChWithdraw **-.503** .220 **-.350** .047 -.180

ChLabile -.278 .058 .027  **.478** -.096

ChAfftoPar  **.329** .029 .173 -.297 -.140

ChAltert  **.440** .035  **.382** .048 -.119

ChVocaliza  **.485** -.098 .299 -.100 -.167

ChInitiati  **.569** -.263  **.401** -.144 .106

ChCreatPla .237 -.230 .153 .036 -.255

ChComplian  **.466** .071 -.089 -.147  **.356**

ChPers .174 -.069 .144 -.019  **.645**

ChAvoidanc -.246 .018 -.020  **.446** .038

ChCompUse  **.454** -.026 .030 .115 .017

DyReciproc  **.835** .217 .015 .094 .052

DyAdaptati  **.750** .081 -.144 .212 -.081

DyFluency  **.757**  **.376** .099 .081 .059

DyConstric **-.723** **-.310** .015 .099 .017

DyTension **-.634** .036  **.610** .027 .115

ChLed  **.731** -.231 -.126  **.308** .094

Pled **-.597** .277 .277 -.267 -.141

F1 = Sensitivity/ Reciprocity/ Child Involvement, F2 = Parent Sensitivity, F3 = Parent Intrusiveness, F4 = Child Withdrawal, F5 = Parent Limit Setting/ Child Compliance
